# Use of MS-GUIDE for identification of protein biomarkers for risk stratification of patients with prostate cancer

**DOI:** 10.1186/s12014-022-09349-x

**Published:** 2022-04-27

**Authors:** Sandra Goetze, Peter Schüffler, Alcibiade Athanasiou, Anika Koetemann, Cedric Poyet, Christian Daniel Fankhauser, Peter J. Wild, Ralph Schiess, Bernd Wollscheid

**Affiliations:** 1 Department of Health Sciences and Technology, Institute of Translational Medicine, Swiss Federal Institute of Technology, ETH Zurich, 8093 Zurich, Switzerland; 2 Swiss Institute of Bioinformatics (SIB), 1015 Lausanne, Switzerland; 3 ETH PHRT Swiss Multi-Omics Center (SMOC), 8093 Zurich, Switzerland; 4 Institute of General and Surgical Pathology, Technical University of Munich, 81675 Munich, Germany; 5 Proteomedix AG, 8952 Schlieren, Switzerland; 6 Clinic of Urology, University Hospital Zurich, University of Zurich, 8091 Zurich, Switzerland; 7 Department of Pathology and Molecular Pathology, University Hospital Zurich, University of Zurich, 8091 Zurich, Switzerland; 8 Dr. Senckenberg Institute of Pathology, University Hospital Frankfurt, 60590 Frankfurt, Germany; 9 Frankfurt Institute for Advanced Studies (FIAS), 60438 Frankfurt, Germany; 10 WILDLAB, University Hospital Frankfurt MVZ GmbH, 60590 Frankfurt, Germany

**Keywords:** Prostate cancer, Risk stratification, ELISA, Parallel reaction monitoring, Machine learning

## Abstract

**Background:**

Non-invasive liquid biopsies could complement current pathological nomograms for risk stratification of prostate cancer patients. Development and testing of potential liquid biopsy markers is time, resource, and cost-intensive. For most protein targets, no antibodies or ELISAs for efficient clinical cohort pre-evaluation are currently available. We reasoned that mass spectrometry-based prescreening would enable the cost-effective and rational preselection of candidates for subsequent clinical-grade ELISA development.

**Methods:**

Using Mass Spectrometry-GUided Immunoassay DEvelopment (MS-GUIDE), we screened 48 literature-derived biomarker candidates for their potential utility in risk stratification scoring of prostate cancer patients. Parallel reaction monitoring was used to evaluate these 48 potential protein markers in a highly multiplexed fashion in a medium-sized patient cohort of 78 patients with ground-truth prostatectomy and clinical follow-up information. Clinical-grade ELISAs were then developed for two of these candidate proteins and used for significance testing in a larger, independent patient cohort of 263 patients.

**Results:**

Machine learning-based analysis of the parallel reaction monitoring data of the liquid biopsies prequalified fibronectin and vitronectin as candidate biomarkers. We evaluated their predictive value for prostate cancer biochemical recurrence scoring in an independent validation cohort of 263 prostate cancer patients using clinical-grade ELISAs. The results of our prostate cancer risk stratification test were statistically significantly 10% better than results of the current gold standards PSA alone, PSA plus prostatectomy biopsy Gleason score, or the National Comprehensive Cancer Network score in prediction of recurrence.

**Conclusion:**

Using MS-GUIDE we identified fibronectin and vitronectin as candidate biomarkers for prostate cancer risk stratification.

**Supplementary Information:**

The online version contains supplementary material available at 10.1186/s12014-022-09349-x.

## Background

Molecular biomarkers derived from genetic variants, transcripts, proteins, protein post-translational modifications, and metabolites play key roles in clinical oncology. Molecular signatures can help predict the likelihood of cancer development or progression and have the potential to detect the disease at an early stage [1, 2]. They can also support treatment decision-making and predict treatment responsiveness [3, 4]. Food and Drug Administration (FDA) approved protein biomarkers include cancer antigen 125 (CA125) in ovarian cancer, carcinoembryonic antigen (CEA) in colon cancer, and prostate-specific antigen (PSA) in prostate cancer [5]. Although numerous single-protein cancer signatures have been known for years [6], a striking discrepancy exists between the efforts to develop new protein biomarkers and the number eventually approved by the FDA. One obstacle is that testing of a protein biomarker candidate in a large-scale validation study poses an organizational and financial challenge that hampers translation into a clinical-grade assay [7].

To overcome limitations in translational biomarker research, various technologies must be combined in a time- and cost-efficient manner. Enzyme-linked immunosorbent assays (ELISAs) are broadly established and routinely used for clinical protein determination because these robust, accurate assays can be produced at low cost and automation provides high throughput. However, an ELISA’s success relies on the availability of highly specific and sensitive monoclonal antibodies. Additionally, it is difficult to multiplex ELISAs, which makes the technology unsuitable for biomarker screening. Modern mass spectrometry (MS)-based proteomics, on the other hand, enables multiple protein marker measurements in parallel with adequate sensitivity and without the need for highly specific antibodies [8]. MS is costly, often semi-quantitative, time-consuming, and technically challenging for routine diagnostic point-of-care applications, however.

**Fig. 1:**
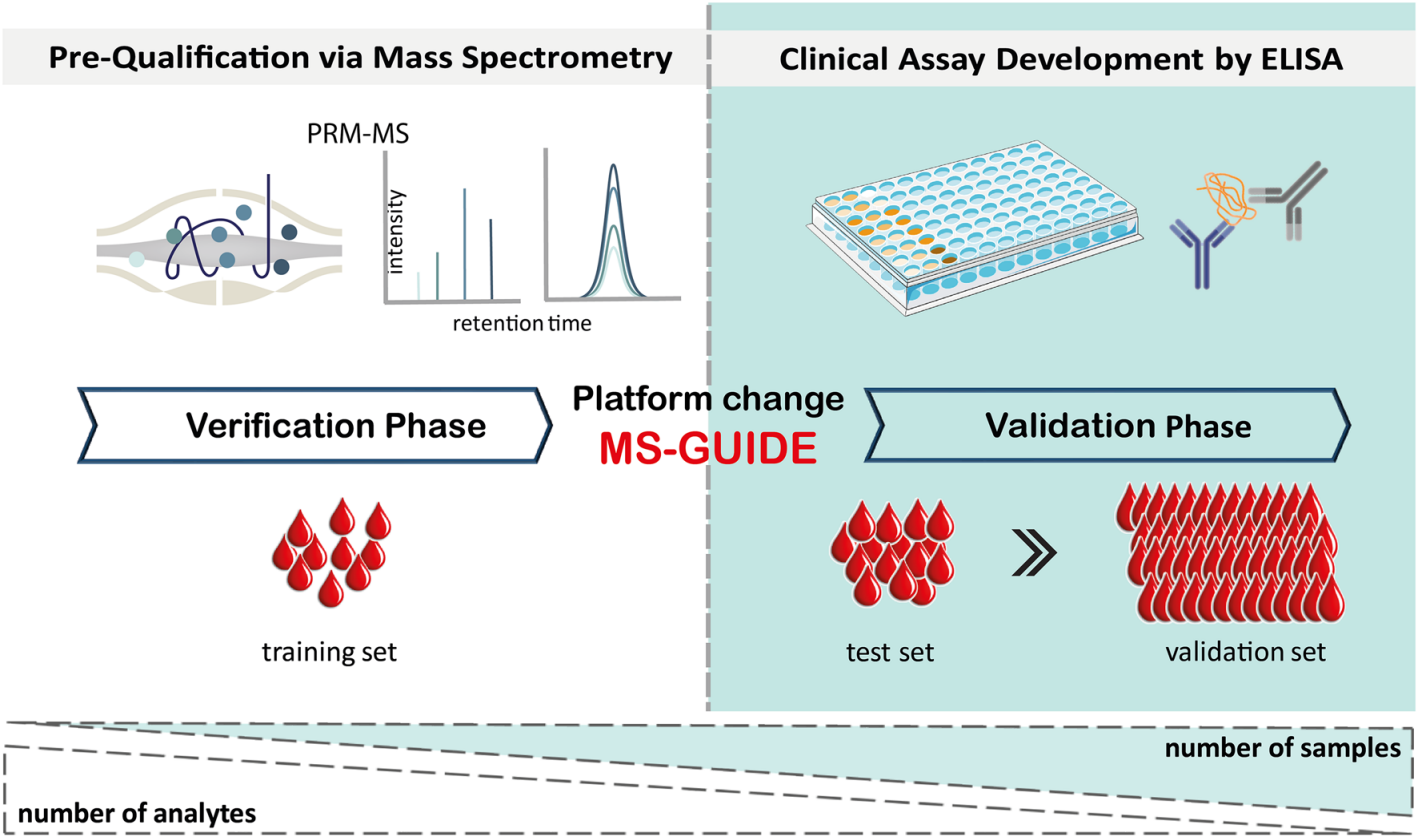
Diagnostic and prognostic biomarker assay development using MS-GUIDE. In a pre-qualification step, PRM-MS is used to screen a high number of potential biomarkers in a multiplexed fashion in samples from a small cohort. For identified candidates, a clinical-level sandwich ELISA is established. ELISAs are highly specific, quantitatively robust, and enable the measurement of hundreds of samples at a time and can therefore be used to validate biomarkers in large cohorts

Here we set out to combine the strengths of analyte throughput with measurement robustness of the ELISA with the power of MS by employing Mass Spectrometry-GUided Immunoassay DEvelopment (MS-GUIDE). MS-GUIDE can be used to evaluate pre-qualified, literature-harvested, potential biomarker candidates, for which no antibodies for sensitive detection in complex samples exist. Parallel reaction monitoring-MS (PRM-MS) is used to evaluate potential protein markers in a highly multiplexed fashion in a medium-sized patient cohort (Fig. 1, training set). ELISAs are then developed for a small subset of candidates with clinical relevance and used for significance testing in larger patient cohorts (Fig. 1, test and validation sets).

We employed MS-GUIDE to identify a prognostic protein stratification panel for men with localized prostate cancer (PCa) with the aim of better defining high-risk disease based on biochemical recurrence-free survival, a strong indicator for disease prognosis. Clinically localized prostate cancer can be controlled by curative radical prostatectomy. Still, around 40 percent of surgically treated men will experience a detectable serum PSA as an unequivocal indicator of cancer progression within 10 years of surgery [9, 10]. These men with biochemical recurrence are at significant risk for clinical cancer progression (metastases). Clinical stage, pretreatment PSA levels, and prostate biopsy Gleason grade are reliable and independent predictors of treatment failure and their combination markedly enhances the ability to predict treatment outcome. However, the usefulness of current models is hampered by their limited predictive accuracy and there is a need to identify novel markers that are specifically linked to the presence of biologically aggressive prostate cancer for improved prediction of outcome in populations with moderately elevated PSA levels [11].

Serum is an attractive source for biomarker discovery as it is easily accessible and contains secreted proteins from tissue that have the potential to reveal pathologic changes in disease. In prostate cancer, secreted proteins have been prioritized as biomarker candidates because even localized prostate cancer is genetically highly variable [12] and biopsy-based sampling is usually random, making accurate stratification of disease based on tissue biopsies alone difficult. However, the sensitivity of protein profiling in serum is compromised by the wide dynamic range of protein concentrations [13]. In the past, various depletion and enrichment strategies have been used to reliably detect proteins in serum even at lower concentrations [14]. For the specific and robust detection and quantification of secreted proteins, which are usually N-glycosylated, enrichment of N-glycosylated proteins by solid Phase Extraction of N-linked Glycopeptides (SPEG) has been successfully applied [15].

**Fig. 2:**
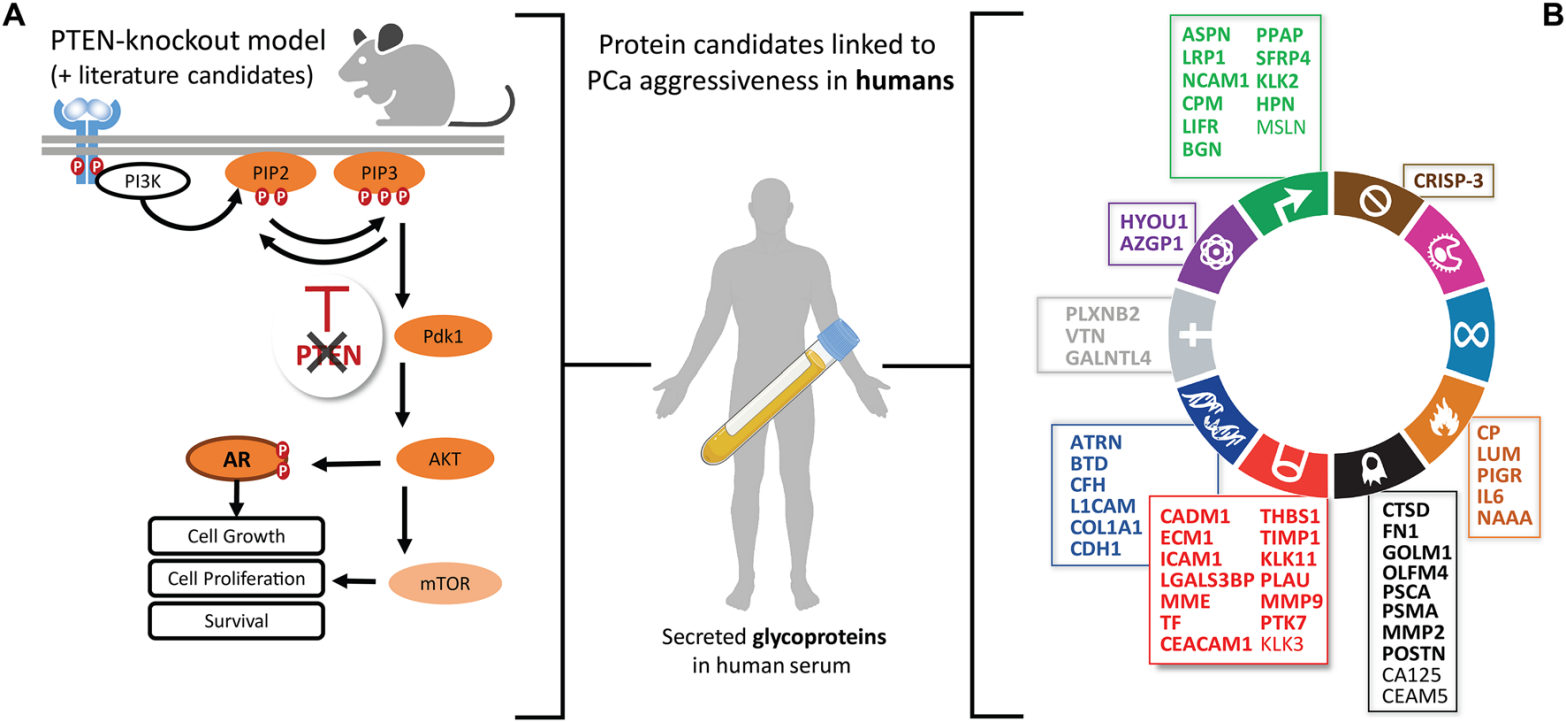
Hypothesis-driven protein marker selection. **A** Most protein biomarker candidates were selected from a previous study of a PTEN-knockout mouse model successfully used to identify diagnostic markers of prostate cancer (9) supplemented with potentially glycosylated and secreted proteins derived from literature. The abundance of these potentially glycosylated and secreted proteins was monitored in serum samples from a prostatectomy cohort. **B** In total, 52 proteins related to various hallmarks of cancer [56] were analyzed in human serum using protein glycocapture. Of these, 48 were proteins with a potential prognostic value in prostate cancer (bold), whereas four additional secreted proteins used in routine diagnostics were monitored as negative controls

Using MS-GUIDE, we mainly screened secreted, N-glycosylated proteins enriched by SPEG for their prognostic potential, which we had previously identified using a *phosphatase and tensin homolog PTEN*-knockout mouse model [16]. PTEN inhibits the PI3K/AKT pathway (Fig. 2A), and loss of the tumor suppressor is one of the best characterized genomic events in prostate cancer [17]. Loss of PTEN is strongly associated with unfavorable oncologic outcomes, making PTEN and its downstream targets potentially useful markers for distinguishing indolent from aggressive disease [18]. Signaling pathway-activating mutations, such as loss of PTEN, result in abundance changes in the cell surface and secretory proteomes of affected tissue [19], and in principle, these changes should be detectable as quantitative biomarker signatures also in serum.

In our study, we identified two secreted extracellular matrix proteins, FN1 (fibronectin; UniProt: P02751/FINC_HUMAN) and VTN (vitronectin; UniProt: P04004/VTNC_HUMAN), which together with PSA predicted biochemical recurrence-free survival statistically better than PSA alone or PSA plus biopsy Gleason score, or the National Comprehensive Cancer Network (NCCN) score. Our MS-derived protein signatures were translated into two high-quality sandwich ELISA assays that were then used to validate the risk stratification potential of the respective proteins in an independent patient cohort. Our clinical-grade ELISAs are readily usable and can support clinicians in their decision on how to manage patients with localized prostate cancer. We expect that MS-GUIDE will become a more widely used approach for efficient and cost-effective biomarker development.

## Methods

### Study design

The Ethics Committee of the Kanton Zurich, Switzerland, approved all procedures involving human material, and all patients gave informed consent (Ref. Nr. StV KEK‐ZH‐Nr. 06/08). Inclusion criteria were the following: initial biopsy; a total PSA concentration between 2 and 10 ng/ml; a negative digital rectal examination; an enlarged prostate with a volume ≥ 35 ml determined by transrectal ultrasound; an available serum sample; and informed consent to use their sample for research purposes. All men with a cancer-positive biopsy outcome underwent subsequent in‐house radical prostatectomy so that the Gleason scores from both the biopsy and the prostatectomy specimen were available. Patients were considered low risk if total PSA was 4–10, tumor stage was pT2, and Gleason score was ≤ 6. Patients were considered high risk if total PSA was above 10, tumor stage was pT3, and Gleason score was ≥ 7. Availability of biochemical recurrence-free survival measures was used as inclusion criteria for validation studies.

Pre-prostatectomy serum samples were included from 118 patients who had been treated with surgery in 2011 at the Department of Urology and the Martini-Klinik, Prostata Cancer Center at the University Medical Center Hamburg-Eppendorf, Germany for whom there was follow-up data (Hamburg prostatectomy cohort, Table 1A) [20]. The 263 pre-prostatectomy serum samples used for validation experiments were available from the ProCOC biobank of the University Hospital Zurich, Switzerland [21]*.* Serum samples were collected from 2008 to 2012 (Table 1B). For long-term storage serum samples were centrifuged at 3800 g for 10 min and kept at − 80 °C. Patient PSA values were measured following surgery and biochemical recurrence was defined as a postoperative PSA ≥ 0.2 ng/ml confirmed by a second determination with a serum PSA ≥ 0.2 ng/ml. Biochemical recurrence-free survival was evaluated over 6–36 months of the patients with yearly reported outcome measurements. Extended information  on the clinical cohorts is provided in Additional file 1: Tables S7 (Hamburg prostatectomy cohort) and S8 (ProCOC).

**Table 1:** Clinical cohort description

A. Hamburg cohort				B. ProCOC cohort			
PSA Density	Gleason	NCCN	Patients	PSA Density	Gleason	NCCN	Patients
< 0.14	≤ 7	1	36	< 0.14	≤ 7	1	47
0.03–0.31	6–7	2	13	0.01–0.31	6–7	2	30
0.02–0.88	6–8	3	55	0.02–0.53	6–9	3	146
0.04–0.98	6–9	4	13	0.03–0.93	7–9	4	40
0.04	6	NA	1	TOTAL			263
TOTAL			118				

A. Summary of clinical parameters of Hamburg (HH) prostatectomy cohort with clinical PSA follow-up of a median of 25 months. B. Summary of clinical parameters of the ProCOC cohort with clinical PSA follow-up of a median of 34 months

### Glycoprotein enrichment of secreted proteins from serum

Glycoproteins were enriched from sera using the protocol published by Zhang et al. [15, 22]. In short, two bovine reference glycoproteins A1AG (Q3SZR3) and FETUB (Q58D62) as well as two human heavy isotope-labeled GST reference proteins, CTSD (P07339) and HYOU1 (Q9Y4L1) were spiked into 50 μl of patient serum at concentrations of 1 pmol/μl for the bovine proteins and 50 ng/μl for the human proteins. The reference proteins were used to evaluate and normalize for intra-sample variation during glycocapture. Glycan-moieties of serum proteins were oxidized using sodium periodate (Sigma-Aldrich). Oxidized proteins were purified using G-10 gel filtration cartridges (Nest Group) and subsequently conjugated overnight to Affi-gel Hydrazide-resin (Bio-Rad). After coupling, samples were washed extensively to remove unbound proteins. Proteins were then digested on beads overnight using a mix of trypsin (Promega) and lysyl endopeptidaseR (Wako). Cleaved non-glycopeptides were washed off the beads, and N-linked glycopeptides were released by the addition of PNGase F (BioConcept). Released peptides were purified before MS via C18 reverse-phase resin (Nest Group). For MS measurements, peptides were solubilized in 100 μl of 0.1% aqueous formic acid (FA) and 2% acetonitrile (ACN). 

### Mass spectrometry measurements

Heavy isotope-labeled reference peptides (SpikeTides™) were obtained from JPT Peptide Technologies GmbH. For spectral library generation, peptides were loaded onto an in-house-packed chromatography column (75-μm inner diameter, New Objective, ReproSil-Pur C18-AQ, 1.9 µm) connected to a nano-flow HPLC combined with an autosampler (EASY-nLC 1000) and a QExactive Plus Orbitrap mass spectrometer (Thermo Fisher Scientific). Injection was with 100% buffer A (99.9% H2O, 0.1% FA). Peptides were eluted at a constant flow rate of 300 nl/min with a 48-min linear gradient from 3 to 35% buffer B (99.9% ACN, 0.1% FA) followed by a 4-min gradient from 35 to 50% buffer B. After the gradient, the column was washed with 98% buffer B. MS1 spectra were acquired at 70,000 resolution (automatic gain control target value 1.0*10e6, maximum injection time 120 ms) to monitor peptide ions in the mass range of 300–1700 m/z, followed by high-energy collisional dissociation (HCD) MS/MS scans (Top15). MS2 spectra were acquired at 35,000 resolution (automatic gain control target value 5.0*10e4, maximum injection time 120 ms) with a quadrupole isolation window of 1.5 m/z. Dynamic exclusion was set to 30 s. Alternatively, heavy peptides for spectral library generation were monitored on an Orbitrap Fusion™ Tribrid™ Mass Spectrometer (Thermo Fisher Scientific) using the universal method (MS1 spectra at 120,000 resolution, automatic gain control target 2.0*10e5, maximum injection time 50 ms, mass range 395–1500 m/z, followed by HCD MS/MS scans using the iontrap at rapid scan rate and a 3 s cycle time (automatic gain control target value 1.0*10e2, maximum injection time 250 ms)).

Formerly glycosylated peptides derived from patient serum samples were analyzed using PRM mode on an Orbitrap Fusion™ Tribrid™ Mass Spectrometer connected to a nano-flow HPLC combined with an autosampler (EASY-nLC 1000, Thermo Fisher Scientific). For each MS run, peptides corresponding to 0.5 μL of patient serum starting material were injected. Peptides were separated by reversed-phase chromatography as described above. Elution was at a constant flow rate of 300 nl/min, the gradient was stepped (75-min linear from 3 to 25% buffer B, 10-min linear from 25 to 50% buffer B). At the end of the gradient, the column was washed twice with 90% buffer B. Mass spectra were acquired at 15,000 resolution (automatic gain control target value 5.0*10e4). Peptide ions were isolated in the mass range of 350–1400 m/z (quadrupole isolation window 1.4), followed by HCD MS/MS scans. The stepped collision energy was set to 27 (± 5%). The maximum injection time was 22 ms, spectra were recorded in profile mode. 

### Mass spectrometry data evaluation

Acquired MS/MS spectra for library generation were converted to mzML using MSconvert. Fragment ion spectra were searched with COMET (2015.01) against UniProtKB/Swiss-Prot (Homo sapiens, release 01406) containing common contaminants. The following search parameters were used for protein identification: (i) peptide mass tolerance was set to 20 ppm, MS/MS mass tolerance to 0.02 Da; (ii) semi-tryptic peptides with up to two missed cleavages were allowed; (iii) carbamidomethylation of cysteine was set as fixed modification; (iv) N deamidation, M oxidation, heavy K, and heavy R were set as variable modifications. Probability scoring was done with PeptideProphet and ProteinProphet of the Trans-Proteomic Pipeline (v4.6.2). For building our spectral library in Skyline, protein identifications were filtered for a false discovery rate of ≤ 1%.

### PRM measurements

For PRM measurements, fragmentation patterns and scheduling windows were first determined based on the heavy isotope-labeled reference peptides spiked into the samples. Scheduling windows were set to 6 min to account for sample-intrinsic retention time shifts during acquisition. The three heavy-labeled GST reference proteins and the two bovine spike-in references were used to assess the efficiency and reproducibility of protein glycocapture and tryptic digest. A mixture of peptides to enable retention time calibration (Biognosys) was used to align for retention time shifts.

Skyline software (v3.7) was used to quantify peptide intensities. Peptide quantitation was based on the summed area of the four most prominent transitions for a given precursor. For protein quantities, the sum of all peptide quantities was taken. Protein quantities were normalized to the bovine A1AG reference to account for sample-specific variation during sample processing. Data was uploaded to PanoramaWeb (https://panoramaweb.org/MS-GUIDE_wlab.url; ProteomeXchange ID PXD016976). Ggplot2 was used to generate a circular heatmap and a violin plot of protein quantitation data.

### ELISA measurements

Sandwich ELISAs were performed as follows: The 96-well MaxiSorp microtiter plates (Nunc) were coated with capture antibodies overnight at 4 °C. The detection antibodies were labeled with biotin according to standard protocols [20]. After coating, plates were blocked for 1 h at room temperature with BSA-Block Solution (Candor Bioscience). Biotinylated detection antibody (R&D Systems) and sample or reference protein were diluted in assay buffer (Low Cross Buffer, Candor Bioscience), added to the plate, and incubated for 60 min at 37 °C. Streptavidin-horseradish-peroxidase (Jackson Immuno Inc.) was added, and samples were incubated for 30 min at 37 °C. After washing, TMB substrate (Enhanced K-Blue, Neogen) was added, and after 30 min at 37 °C, the reaction was stopped with 1 M HCl solution. Plates were washed using a Hydrospeed-Washer from TECAN. Sample readout was achieved at 450 nm with a TECAN F50 Infinite reader using Magellan 7.0 software.

### Machine learning and statistical data analysis

Training of the models was conducted in R (https://www.r-project.org/). Training data were pre-processed using the following steps: in the MS training data, individual outliers were detected and removed using the *extremevalues* package (https://cran.r-project.org/web/packages/extremevalues/index.html) [23]. Only feature candidates with measurements in over two-thirds of samples were used. Missing values were imputed using the *mice* package [24] with the random forest-based method. No data scaling was encountered as random forests are scale-invariant. Models were trained using the *randomForest* package [25], with 200 trees per forest unless indicated differently. Random forests were trained on the two classes, recurrence vs. no recurrence, in all experiments. To model patient groups with biochemical recurrence-free survival, patients were stratified into a low risk and a high risk group using a cutoff of the predicted score such that sensitivity and specificity are maximized. Several measures were included to avoid overfitting. First, we trained with a relatively high number of trees to reduce the chance of overfitting by training on different dataset splits (including cross-validation). Second, instead of relying on the best-trained model, we averaged the features over a group of statistically indistinguishable models. Finally, we included the two best features VTN and FN1 in the final model. On the ELISA data set, similar pre-processing steps were applied. Reported p-values were corrected for multiple testing using the Bonferroni method unless indicated differently. Plots and statistics were created using the *survival* and *pROC* packages [26]. The code is available at https://github.com/jetic83/MSGUIDE.

## Results

### Biomarker pre-qualification via PRM-MS identifies two marker proteins for prostate cancer aggressiveness

A retrospective study on previously biobanked samples was performed to identify pre-selected serum proteins that discriminate aggressive from non-aggressive prostate cancer more accurately than PSA density (PSA normalized to prostate volume) and/or biopsy (Bx) Gleason score. PRM-MS was used to quantify secreted tissue glycoproteins that had a known connection to prostate cancer development (hypothesis-driven marker selection) ([16, 27–31] (Additional file 1: Table S1) in samples from a training cohort (pre-qualification, Fig. 1). Most protein biomarker candidates were selected based on our previous analysis of a *phosphatase and tensin homolog PTEN*-knockout mouse model [16]. It was shown in the past that the loss of PTEN produces abundance changes on the cellular surfaceome and secreted proteomes of the affected tissues and that these abundance changes are also partially detectable in serum [8, 16]. Human orthologs from proteins affected by the *PTEN*-knockout in mice were shown to be equally detectable in serum from prostate cancer patients [16]. In total, we investigated 30 proteins that have been associated with prostate cancer development or progression through the *PTEN*-knockout mouse model and whose human orthologs were reported to be detectable and affected in serum. Four proteins from the Oncotype DX prostate cancer panel [27] and an additional 14 potential markers of prostate cancer aggressiveness known to be secreted or associated with the plasma membrane, as shown in the literature [28–31], were also included (Additional file 1: Table S1). Candidate proteins were prioritized based on evidence for peptide detectability in plasma. Four additional glycosylated protein markers that are used in routine diagnostics were chosen as unrelated controls in our study. In total, 52 proteins were monitored using PRM. Their potential role in prostate cancer development and association to different hallmarks of cancer is depicted in Fig. 2 (Baker et al. 2017) (also see Additional file 1: Table S1).

**Fig. 3:**
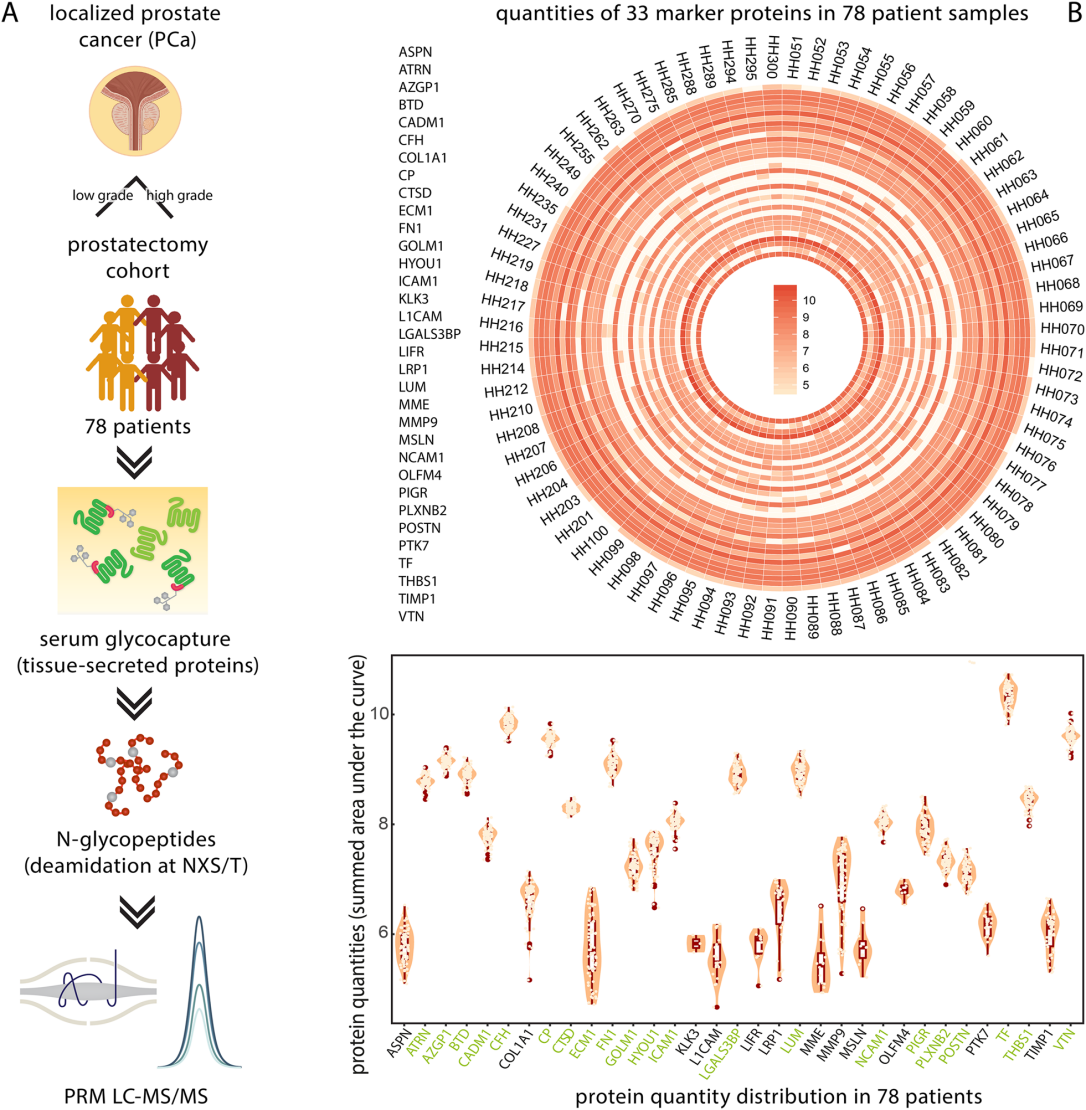
Protein marker pre-qualification by mass spectrometry. **A** Potential prognostic biomarkers of prostate cancer were monitored in a prostatectomy cohort consisting of 38 patients with low-grade (NCCN 1, 2) and 40 patients with high-grade disease (NCCN 3, 4). Serum protein glycocapture was performed [15] and deamidated, formerly glycosylated peptides were monitored using PRM-MS. **B** From our list of 52 marker proteins, 33 were detected and quantified in our training cohort. The heatmap illustrates the intensity distribution of protein quantities over the cohort from ASPN (outside of the circle) to VTN (inside of the circle). Violin plots visualize data distribution and probability density. Distribution median and quartiles are shown in red. Single protein values are indicated by dots. Proteins that were used for machine learning are designated in green

In our PRM study, sera from 78 individuals collected before undergoing prostatectomy were included (Hamburg cohort, Additional file 1: Table S2). The staging was NCCN = 1 for 29 patients, NCCN = 2 for 9 patients, NCCN = 3 for 23 patients, and NCCN = 4 for 17 patients. NCCN 1 and 2 were considered low-grade (38 patients), NCCN 3 and 4 high-grade disease (40 patients). Samples were subjected to solid-phase extraction of N-glycopeptides [15]. The enriched, deamidated N-glycopeptides harboring an NXS/T motif were quantified on a Fusion Orbitrap mass spectrometer using targeted proteomics and PRM-MS (Fig. 3A). To define retention time windows and to confirm the spectral identification of endogenous peptides, a heavy isotope-labeled synthetic peptide library was generated (Additional file 1: Table S3). In addition to our target list of 52 proteins, we monitored bovine Alpha-1 Acid Glycoprotein (A1AG) and bovine Fetuin B (FETUB), which were added to the sample prior to glycocapture as processing references. Also, a mixture of peptides to enable retention time calibration was recorded. In samples from the prostate cancer patient training cohort, we monitored for a total of 151 precursors (Additional file 1: Table S4). Of these, we were able to quantify A1AG, FETUB, peptides for retention time calibration, and 68 precursors, corresponding to 33 candidate biomarkers (Fig. 3B).

Proteins identified in all samples with no more than one-third missing values were selected as candidates for ELISA development. This resulted in a list of 21 potential biomarker candidates: ATRN, AZGP1, BTD, CADM1, CFH, CP, CTSD,  ECM1, FN1, GOLM1, HYOU1, ICAM1, LGALS3BP, LUM, NCAM1, PIGR, PLXNB2, POSTN, TF,  THBS1, and VTN (Fig. 3B, proteins annotated in green). This list was subjected to machine learning using a random forest classifier algorithm for dedicated feature selection by predicting risk groups.

### MS data classification and model development

**Fig. 4:**
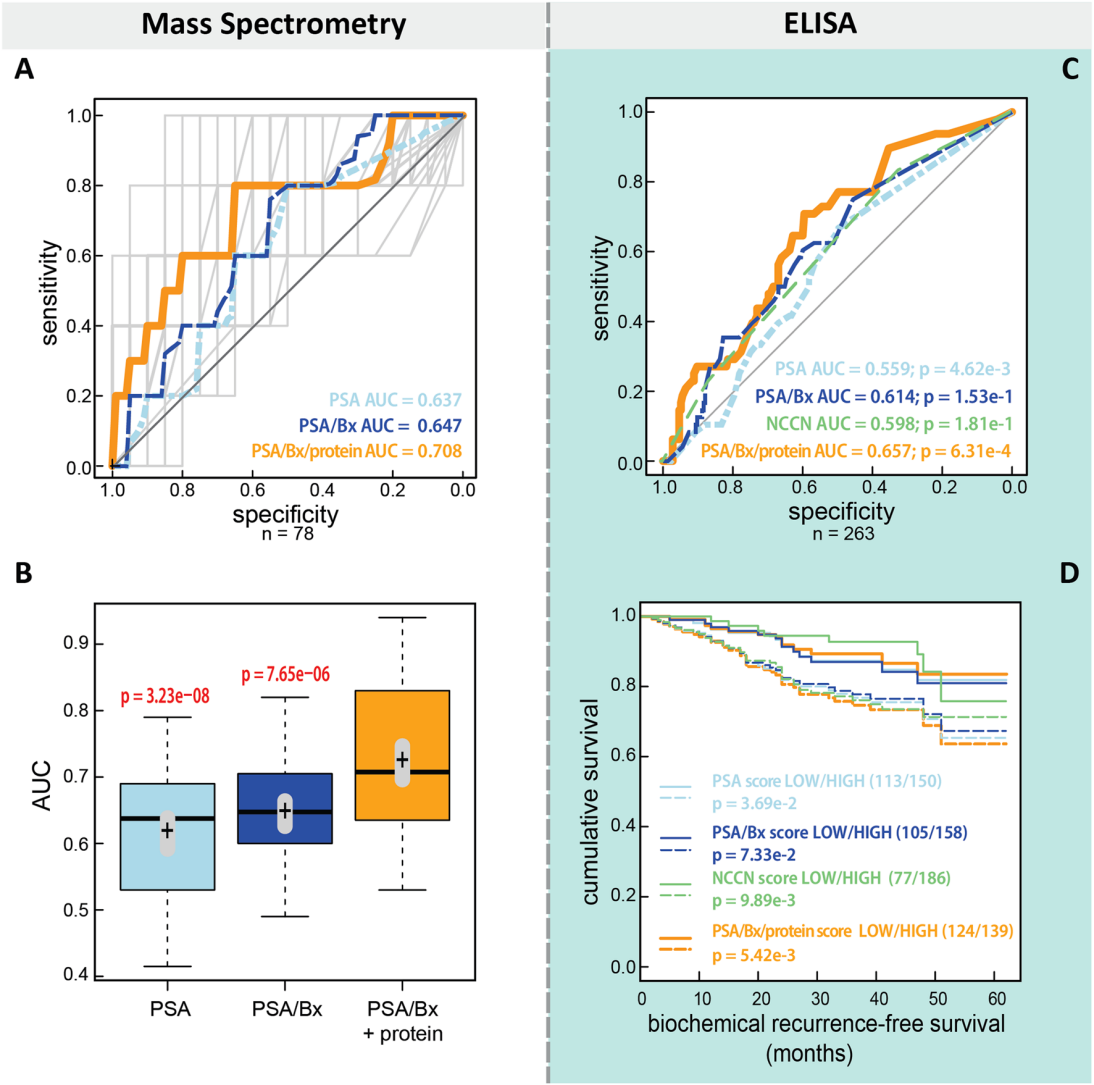
Predictive ability of FN1 and VTN ELISA data concerning recurrence-free survival. **A** AUCs for the Hamburg cohort based on FN1 and VTN levels determined using MS (n = 78). Shown are median AUCs of 50-fold cross-validation (grey) of our model using FN1 and VTN (protein) plus PSA plus Gleason score (Bx; orange) versus PSA alone (light blue) and PSA plus Bx (dark blue). **B** Boxplots of our model for the Hamburg cohort with protein levels determined by MS (PSA/Bx + protein) compared to PSA alone and PSA plus Bx. Each box indicates min, 25%-quantile, median (black line), 75%-quantile, max, mean (black cross), and std (gray bar). Statistics: paired t-test, corrected for multiple testing with the Benjamini and Hochberg method [57]. **C** Prediction of 5-year biochemical recurrence-free survival for the validation ProCOC (n = 263) with our model based on the protein signature determined by ELISA (FN1, VTN, PSA, and Bx, orange) versus PSA alone (light blue), PSA plus Bx (dark blue), and NCCN alone (green). Statistics: DeLong test for ROCs. **D** Kaplan–Meier plots for recurrence-free survival of ProCOC (n = 263) stratified based on PSA (light blue), PSA plus Bx (dark blue), NCCN alone (green), or our score (orange lines). Statistics: Likelihood ratio test

To identify significant features of prostate cancer aggressiveness, we applied a random forest classifier algorithm for variable ranking and subsequent selection. Random forest classification is particularly well-suited for this application, as this classification approach does not assume that the data are linearly separable. In an exhaustive search, all up to 5-plex combinations were built of the 21 identified marker candidates together with PSA, resulting in 27,895 different models. Each of these marker combinations was validated with a random forest classifier of 200 trees by 50-fold bootstrapped cross-validation [32]. We ranked each model by the median area under the receiver operating characteristic curve (AUC) over the 50-fold bootstrapped cross-validations, thereby identifying the models able to predict prostate recurrence significantly better than PSA alone and better than PSA plus biopsy Gleason score (Additional file 3: Fig. S1A). The top 62 models were statistically not significantly different from each other (paired Student’s t-test, Bonferroni corrected for multiple testing). The predictor distribution of these “statistically equivalent” models was stable regardless of whether the top 62 (Additional file 3: Fig. S1B) or top 100 models were included. Fibronectin (FN1) and Vitronectin (VTN) are top drivers of survival prognosis prediction together with the cell adhesion molecule CADM1. The univariate expression of FN1 and VTN (Additional file 3: Fig. S2) supports the predictive potential of the two candidates in patients with and without recurrence. To generate a final model, we added the protein quantity information of FN1 and VTN to the baseline prediction of PSA and Gleason score (Bx). We chose this two-protein signature as it was the most stable predictor of prostate cancer aggressiveness and was selected in all 62 top models. The median AUC of our combined model (AUC = 0.708, 95% CI [0.675, 0.740]) predicts biochemical recurrence-free survival significantly better than PSA alone (AUC = 0.637, CI [0.560, 0.655], paired t-test p = 3.23e−08) or PSA plus biopsy Gleason score (AUC = 0.647, CI [0.605, 0.670], p = 7.65e−06, Fig. 4A, B). Thus, this model stratifies high-risk from low-risk prostate cancer patients better than current clinical models (Additional file 3: Fig. S3).

### MS-based prognostic marker panel is transferable to an antibody-based ELISA platform

We next developed sandwich ELISAs for FN1 and VTN. Monoclonal antibodies were generated by immunization of mice using native FN1 and VTN (Additional file 2: Materials and Methods). In total, 32 antibody pairs were tested in a label-free biosensor-based assay essentially as previously described [33]. From these antibody pairs, we established two clinical-grade ELISAs with affinities (KDs) in the range of 10^–10^ M (Additional file 1: Table S5). The dynamic range was between 4.8 and 1120 µg/ml, intra and inter-CV of the immunoassays were respectively below 2.6% and 8.1% (Additional file 1: Table S6).

To validate the use of FN1 and VTN as prognostic biomarkers for prostate cancer, we used the newly established ELISAs to analyze samples from 118 patients from the Hamburg prostatectomy cohort of patients who had been treated with prostatectomy and for who long-term biochemical recurrence-free survival data are available (Table 1A). For 78 patient samples both PRM-MS and ELISA were performed. To generate a model, the random forest classifier was trained on FN1, VTN, PSA, and biopsy Gleason score from all 118 patients from the Hamburg cohort with 200 trees. Comparison models were trained in the same way using PSA only or PSA together with biopsy Gleason score. The model that included FN1 and VTN predicts 5-year biochemical recurrence-free survival for this cohort with a training AUC of 0.96 (95% CI [0.92, 0.99], p = 4.48e−10), which is better than for PSA alone (AUC = 0.87, CI [0.76, 0.98], p = 0.0719), PSA plus Gleason score (Bx) (AUC = 0.90, CI [0.81, 0.98], p = 0.139), or NCCN score (AUC = 0.73, CI [0.62, 0.84],_,_ p = 5.3e−5) (Additional file 3: Fig. S4, all training performance).

### Validation of FN1 and VTN as prognostic markers in an independent cohort

Finally, we set out to validate our prognostic protein signature in an independent prostatectomy cohort, the Prostate Cancer Outcomes Cohort (ProCOC). Serum and long-term survival data are available for the 263 prostate cancer patients in this cohort (Table 1B; Additional file 1: Table S7) [21]. The benchmarking on this independent patient cohort revealed that the combination of the two-protein signature together with PSA alone or with PSA plus Gleason score Bx significantly outperforms the state-of-the-art measures for prostate cancer aggressiveness. The combination of all parameters yielded an AUC of 0.66 (95% CI [0.58, 0.74], Fig. 4C, p = 6e−4) when predicting biochemical recurrence-free survival, whereas PSA alone, PSA and Gleason score, and NCCN score alone resulted in AUCs of 0.56 (CI [0.48, 0.64], p = 4e−3), 0.61 (CI [0.53, 0.70], p = 0.153), and 0.60 (CI [0.52, 0.68], p = 0.181), respectively (Fig. 4C). By applying a cutoff with maximum sum of sensitivity and specificity to our dataset, we separated our cohort into low-risk (below the cutoff) and high-risk cases (above the cutoff). A Kaplan–Meier estimate shows that our model separated the low-risk patient group from the high-risk patient group significantly better (n_low_ = 124, n_high_ = 139, p = 5e−3) than the reference models PSA (n_low_ = 113, n_high_ = 150, p = 4e−2) and PSA plus Gleason score (n_low_ = 105, n_high_ = 158, p = 7e−2), although not as well as the NCCN score (n_low_ = 77, n_high_ = 186, p = 10e−3) (Fig. 4D).

## Discussion

Despite extensive protein biomarker discovery efforts by industry and academia, few clinical diagnostic tests meaningfully impact cancer clinical care [34, 35]. Newly discovered biomarkers frequently show a weak clinical performance concerning their sensitivity and/or specificity or fail validation in independent cohorts for statistical reasons. Mass spectrometry-based quantitation assays are difficult to develop for point-of-care use and their application requires significant technical expertise. However, the simultaneous quantitative screening of a large number of candidate biomarkers by PRM-MS allows for the discovery of new potential biomarkers irrespective of the availability of established immunoassays. Clinical-grade ELISAs, on the other hand, are highly sensitive and robust, but they require specific antibodies. The establishment of ELISAs is costly and is only feasible for a limited number of proteins [36]. Here, we showed that the combination of a hypothesis-driven targeted mass spectrometry screening approach in the context of an adequately sized patient cohort and powerful statistics is a versatile tool to identify predictive signatures of disease. These MS-derived marker signatures were then translated into clinical-grade ELISAs that can be applied to robustly validate marker panels in large, independent patient cohorts in a reasonable timeframe. Here we demonstrated the utility of this knowledge-based, two-step validation and verification strategy that we call MS-GUIDE in prostate cancer biomarker discovery. The strategy should prove generally applicable in disease settings other than cancer.

Gene expression profiling has been shown to be useful in predicting clinical outcomes and treatment responses in prostate cancer [37]. Tissue-based genomic classifiers that monitor gene expression panels such as Decipher™ [38], Oncotype DX® [39], and Prolaris® [40], provide useful diagnostic and prognostic information. However, they also have some limitations: Prostate cancer is genetically complex, and mutational processes during tumor development result in intra- and inter-tumor heterogeneity even in localized prostate cancer. This genetic variability makes it difficult to accurately diagnose prostate cancer based on a limited number of tissue biopsies. Moreover, there is a considerable risk of undersampling, which reduces the significance of tissue-based diagnostic tools. Additionally, proteomic subtypes of prostate tumors are only weakly related to their genomic subtypes [41], which in turn stresses the importance of protein quantity information in biomarker panels. Our prognostic protein panel specifically monitors tissue-secreted glycoproteins present in serum. Secreted proteins are involved in cell–cell communication and differentiation and often reflect the developmental and diseased state of a cell [42] as illustrated by preponderance glycoproteins in FDA-approved protein biomarkers [43–45].

Using MS-GUIDE, we derived a new prognostic protein signature for localized prostate cancer. This signature may be helpful in the pre-operative setting to stratify between men with indolent and lethal disease, or could help to reduce overtreatment in men with indolent prostate cancer as well as lead to the identification of men with lethal disease, who eventually require more intense treatment (e.g. longer duration of androgen deprivation therapy together with dose intensified radiotherapy). FN1 and VTN, which we identified as prognostic signature proteins, are components of the extracellular matrix (ECM). The ECM is a major structural component of the tumor microenvironment, and FN1 and VTN are part of the group of glycoproteins that make the ECM a cohesive network linking cells together with other structural components [46]. FN1 was found to be a marker for epithelial-mesenchymal transition-driven cancer stemness [47]. VTN was previously identified as a serum-derived component that drives the differentiation of prostate cancer stem cells, which is in turn related to tumorigenesis [48, 49]. Both proteins function in cell adhesion and spreading and are involved in platelet activation, signaling, and cell aggregation. We found that the abundance levels of these two proteins are inversely correlated with disease aggressiveness. FN1 was recently also found to be downregulated in several tissue carcinomas [50], suggesting a general contribution of platelet activation and ECM organization to tumorigenesis. In fact, many malignancies share dysregulations in the context of molecular pathways that lead to similar systematic disorders and common responses to therapy [51]. Therefore, FN1 and VTN might also be translatable to other cancer types or disease settings. The addition of CADM1, which like FN1 and VTN was a driver of survival prognosis prediction, to this panel might boost its predictive significance. CADM1 belongs to a family of genes involved in the maintenance of cell–cell adhesion in a variety of human epithelial tissues. Previous studies have shown that decreased expression of CADM1 is correlated with tumor aggressiveness and progression in numerous types of cancer [52–54].

We cannot exclude that through our preselection strategy and focus on N-glycosylated proteins, we may have overlooked biomarkers that could also play a role in PCa progression. Also, even though we used heavy reference peptides for assay development, PRM parameters could be further optimized with respect to LOD in serum. However, the MS-GUIDE strategy per se is generally applicable and can easily be translated to other experimental setups and marker preselection schemes in the future to address these limitations.

## Conclusions

In the context of precision diagnostics, there is a need for better and more reliable protein markers to provide actionable information for disease diagnosis, prognosis, prediction, monitoring and stratification to guide treatment decisions [55]. In this work, we present the MS-GUIDE systematic approach for the identification, selection, and validation of potential marker proteins, starting from mass spectrometry-based peptide screening in serum to the development of ELISA assays that can be used in routine diagnostics. Our derived two-protein signature together with PSA stratifies patients with localized prostate cancer 10% better than current gold standards of PCa diagnostics. Our marker panel could be used to complement standard risk stratification schemes based on staging, grading, and PSA measurements, providing patients with localized prostate cancer and their physicians with a tool to assist in treatment decision making.

## Supplementary Information

**Additional file 1: Table S1.** Pre-selected protein candidates that were monitored using PRM-MS. **Table S2.** A subpopulation of the Hamburg (HH) cohort (pre-operation serum was used for protein glycocapture and PRM-MS). **Table S3.** Sequ ences of heavy-isotope labeled synthetic peptides that were used for spectral library generation. **Table S4.** Peptide isolation list for PRM measurements of glycocapture. **Table S5.** Antibody affinities of generated ELISA sandwich pairs. **Table S6.** Dynamic range and precision of developed immunoassays. **Table S7.** Clinical data of Hamburg cohort patients used for random forest classification. **Table S8.** Clinical data of ProCOC patients used for protein signature validation. **Table S9.** ELISA data used for model generation (Hamburg) and validation (ProCOC).

**Additional file 2. **Materials and methods.

**Additional file 3: Fig. S1.** Random forest classification of mass spectrometry derived protein quantities. **(A)** Median AUCs (horizontal black lines in boxes), mean AUCs (black crosses), and std (gray bars) for the top 10 random forest models (orange) compared to PSA alone (light blue) and PSA plus biopsy Gleason score Bx (dark blue). Each model consists of PSA plus the shown peptides, and each was tested in a 50-fold bootstrapped cross-validation experiment to predict biochemical recurrence-free survival. In total, there were 27,895 tested models (all combinations of one to five peptides). Of these, 21,151 were better than PSA or better than PSA plus Gleason score, and 62 models were statistically not significantly different from the best model. **(B)** Differentiating proteins in the 62 statistically identical models. **Fig. S2.** Univariate expression of FN1 and VTN in patients with and without recurrence. The univariate expression supports the use of the two proteins in differential models, but their discriminative power alone is not high enough. **Fig. S3.** Cross-validated Kaplan–Meier plot for Hamburg cohort based on MS data (n = 78). Kaplan–Meier curves of all 50 folds (gray) of our protein model (VTN + FN1) with PSA and biopsy Gleason score (Bx), and the median (orange, p = 0.183). The median plot stratifies the patient groups better than PSA alone (light blue, p = 0.358) and better than PSA plus Gleason score (dark blue, p = 0.229). The p-values indicate likelihood-ratio tests. The patient data of each of the 50 bootstrapped folds were used to train a model, and the model was then applied to the leftover samples to predict sample scores. The scores were binarized into high and low risk based on a cutoff on the training AUC where specificity and sensitivity were maximized. **Fig. S4.** ELISA training error. Trained on Hamburg cohort data (n = 118), the model predicts 5-year biochemical recurrence-free survival on the same Hamburg cohort data with an AUC of 0.956 (orange, 95% CI [0.92, 0.99], p = 4.48e-10), which is better than PSA alone (light blue, AUC = 0.872, CI [0.76, 0.98], p = 0.0719), PSA plus Gleason score (Bx) (dark blue, AUC = 0.898, CI [0.81, 0.98], p = 0.139), or NCCN (green, AUC = 0.73, CI [0.62, 0.84], p = 5.3e-5). Gray line indicates chance level.

## Data Availability

The PRM datasets supporting the conclusions of this article are available in PanoramaWeb (https://panoramaweb.org/MS-GUIDE_wlab.url, ProteomeXchange ID PXD016976)). All other datasets supporting the conclusions of this article are included within the article and its additional files.

## Abbreviations

AUC: Area under the curve
Bx: Biopsy
CADM1: Cell Adhesion Molecule
ELISA: Enzyme linked immunosorbent assay
FDA: US Food and Drug Administration
FN1: Fibronectin
GS: Gleason score
MS: Mass spectrometry
MS-GUIDE: Mass Spectrometry-GUided Immunoassay DEvelopment
NCCN: National Comprehensive Cancer Network
PSA: Prostate-specific antigen
PRM: Parallel reaction monitoring
VTN: Vitronectin
