# Supplementary material for: Use of MS-GUIDE for identification of protein biomarkers for risk stratification of patients with prostate cancer
Source: Clin Proteomics. 2022 Apr 27;19:9. doi: 10.1186/s12014-022-09349-x (PMC9044739; doi:10.1186/s12014-022-09349-x)
Supplement: Supplementary file 2 — Additional file 2. Materials and methods. [file 12014_2022_9349_MOESM2_ESM.docx]

**Materials and Methods**

**Sandwich ELISA assay development**

The generation of monoclonal antibodies was outsourced to an established antibody manufacturer (BioGenes). Immunization was performed in mice using native FN1 and VTN (Creative BioMart). For each antibody, hybridoma cell lines were generated. Binding affinities of antibodies were determined using surface plasmon resonance and Octet-red based biolayer interferometry as described elsewhere[[33]](https://paperpile.com/c/aDTAip/dZbqK). Affinity measurements were carried out for 12 antibodies against FN1 and 20 antibodies against VTN. Antibodies were coupled via amine coupling to biosensor tips. For the determination of the dissociation curve, the biosensor tip containing the coupled antibody complexes was dipped into a well with Low Cross buffer (Candor). When the complexes began to dissociate, K_on_ and K_off_ were determined (Table S5). Measurements were carried out at 37 °C. Data fitting was performed using a 1:1 Langmuir model with Analysis Software V 8.2.0.7 (FortéBio) for the Octet data and Biacore X100 Evaluation Version 2.0.1 Plus Package (GE Healthcare). Sandwich ELISAs were validated by assessing sensitivity, linear range (parallelism, Lower limit of quantification LloQ, Upper limit of quantification UloQ), precision (intra/inter coefficient of variation CV, homogeneity) (table S6), interference substances (HAMA, RF, intralipid, bilirubin, hemoglobin, human serum albumin), robustness, and component stability at 4 °C and 25 °C.
