# Supplementary material for: Use of MS-GUIDE for identification of protein biomarkers for risk stratification of patients with prostate cancer
Source: Clin Proteomics. 2022 Apr 27;19:9. doi: 10.1186/s12014-022-09349-x (PMC9044739; doi:10.1186/s12014-022-09349-x)
Supplement: Supplementary file 3 — Additional file 3: Fig. S1. Random forest classification of mass spectrometry derived protein quantities. (A) Median AUCs (horizontal black lines in boxes), mean AUCs (black crosses), and std (gray bars) for the top 10 random forest models (orange) compared to PSA alone (light blue) and PSA plus biopsy Gleason score Bx (dark blue). Each model consists of PSA plus the shown peptides, and each was tested in a 50-fold bootstrapped cross-validation experiment to predict biochemical recurrence-free survival. In total, there were 27,895 tested models (all combinations of one to five peptides). Of these, 21,151 were better than PSA or better than PSA plus Gleason score, and 62 models were statistically not significantly different from the best model. (B) Differentiating proteins in the 62 statistically identical models. Fig. S2. Univariate expression of FN1 and VTN in patients with and without recurrence. The univariate expression supports the use of the two proteins in differential models, but their discriminative power alone is not high enough. Fig. S3. Cross-validated Kaplan–Meier plot for Hamburg cohort based on MS data (n = 78). Kaplan–Meier curves of all 50 folds (gray) of our protein model (VTN + FN1) with PSA and biopsy Gleason score (Bx), and the median (orange, p = 0.183). The median plot stratifies the patient groups better than PSA alone (light blue, p = 0.358) and better than PSA plus Gleason score (dark blue, p = 0.229). The p-values indicate likelihood-ratio tests. The patient data of each of the 50 bootstrapped folds were used to train a model, and the model was then applied to the leftover samples to predict sample scores. The scores were binarized into high and low risk based on a cutoff on the training AUC where specificity and sensitivity were maximized. Fig. S4. ELISA training error. Trained on Hamburg cohort data (n = 118), the model predicts 5-year biochemical recurrence-free survival on the same Hamburg cohort data with an AUC of 0.956 (orange, 95% [file 12014_2022_9349_MOESM3_ESM.docx]

#### Additional Figures


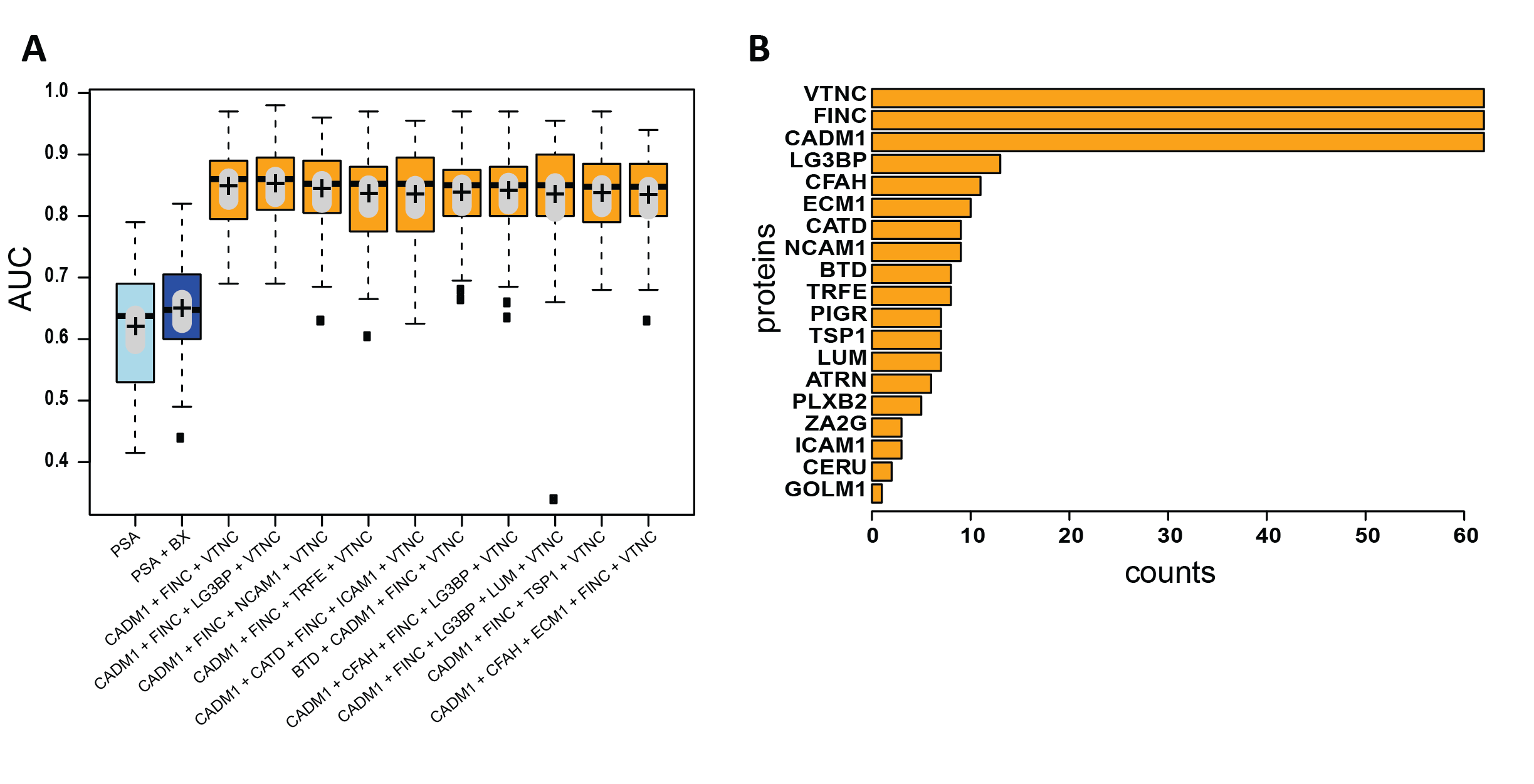


##### Fig. S1. Random forest classification of mass spectrometry derived protein quantities.

**(A)** Median AUCs (horizontal black lines in boxes), mean AUCs (black crosses), and std (gray bars) for the top 10 random forest models (orange) compared to PSA alone (light blue) and PSA plus biopsy Gleason score Bx (dark blue). Each model consists of PSA plus the shown peptides, and each was tested in a 50-fold bootstrapped cross-validation experiment to predict biochemical recurrence-free survival. In total, there were 27895 tested models (all combinations of one to five peptides). Of these, 21151 were better than PSA or better than PSA plus Gleason score, and 62 models were statistically not significantly different from the best model. **(B)** Differentiating proteins in the 62 statistically identical models.


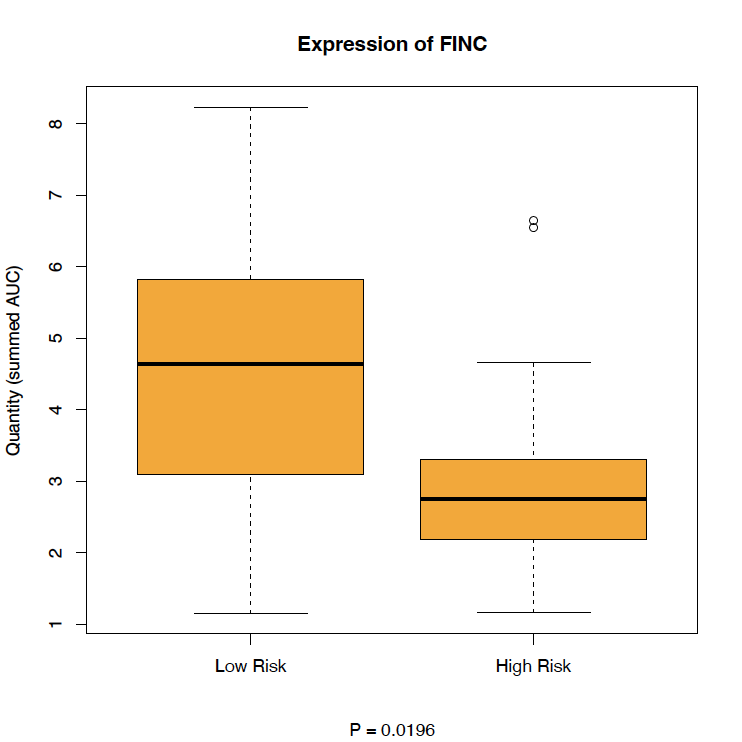

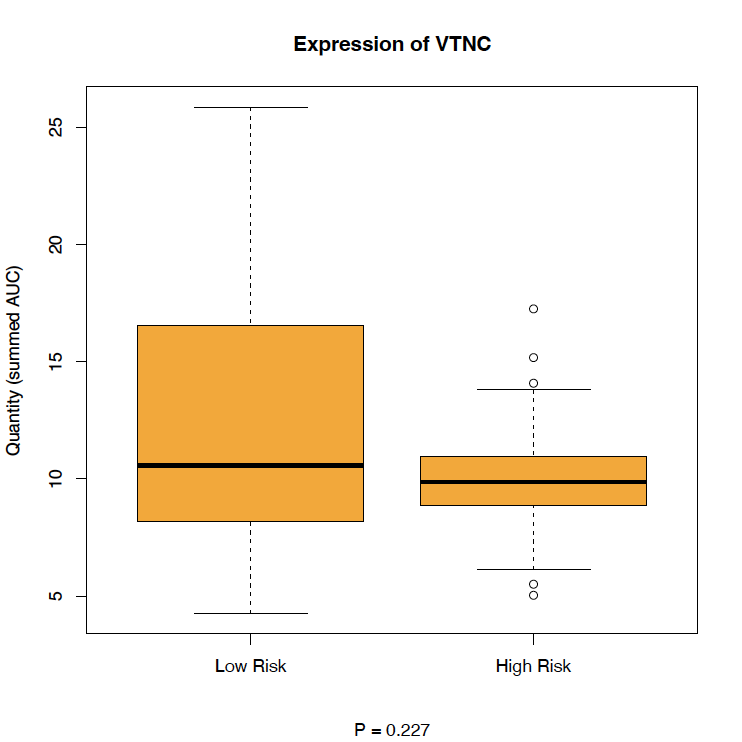


##### Fig. S2. Univariate expression of FN1 and VTN in patients with and without recurrence.

The univariate expression supports the use of the two proteins in differential models, but their discriminative power alone is not high enough.

**
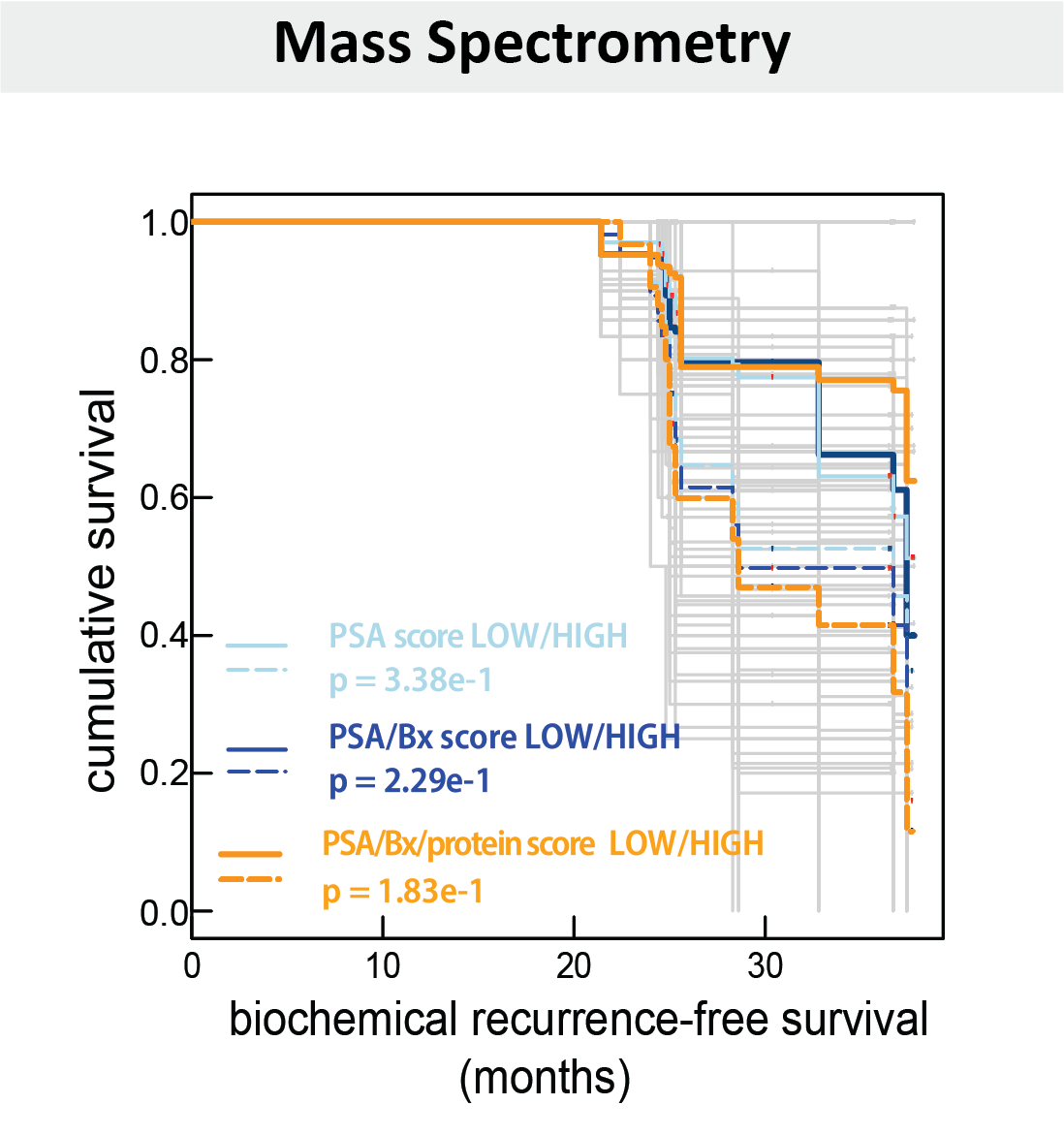
**

##### Fig. S3. Cross-validated Kaplan-Meier plot for Hamburg cohort based on MS data (n=78).

Kaplan-Meier curves of all 50 folds (gray) of our protein model (VTN + FN1) with PSA and biopsy Gleason score (Bx), and the median (orange, p = 0.183). The median plot stratifies the patient groups better than PSA alone (light blue, p = 0.358) and better than PSA plus Gleason score (dark blue, p = 0.229). The p-values indicate likelihood-ratio tests. The patient data of each of the 50 bootstrapped folds were used to train a model, and the model was then applied to the leftover samples to predict sample scores. The scores were binarized into high and low risk based on a cutoff on the training AUC where specificity and sensitivity were maximized.


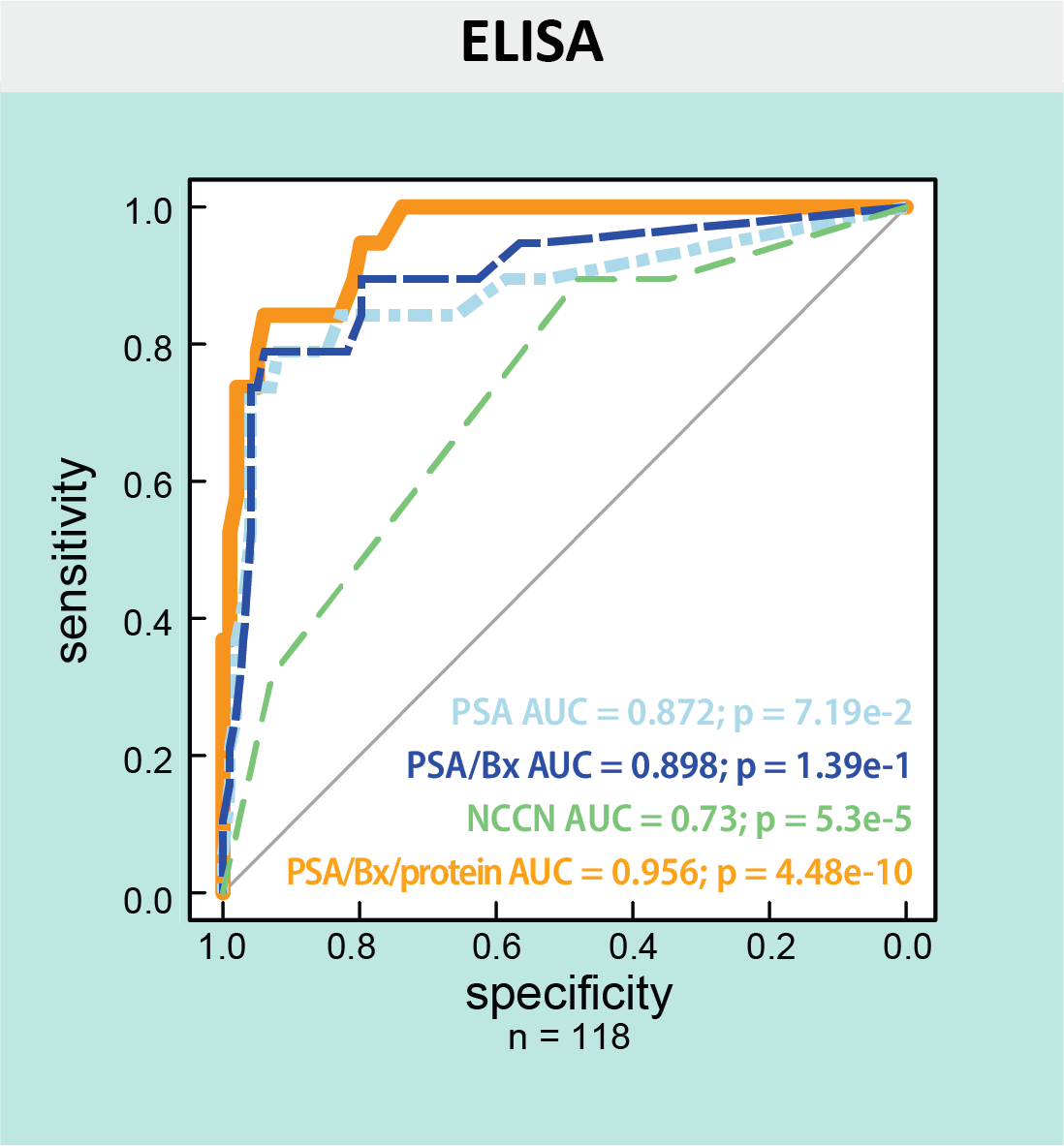


##### Fig. S4. ELISA training error.

Trained on Hamburg cohort data (n=118), the model predicts 5-year biochemical recurrence-free survival on the same Hamburg cohort data with an AUC of 0.956 (orange, 95% CI [0.92, 0.99], p = 4.48e-10), which is better than PSA alone (light blue, AUC = 0.872, CI [0.76, 0.98], p = 0.0719), PSA plus Gleason score (Bx) (dark blue, AUC = 0.898, CI [0.81, 0.98], p = 0.139), or NCCN (green, AUC = 0.73, CI [0.62, 0.84], p = 5.3e-5). Gray line indicates chance level.
